# Different Mi-2 Complexes for Various Developmental Functions in *Caenorhabditis elegans*

**DOI:** 10.1371/journal.pone.0013681

**Published:** 2010-10-27

**Authors:** Myriam Passannante, Claude-Olivier Marti, Catherine Pfefferli, Paolo S. Moroni, Stéphanie Kaeser-Pebernard, Alessandro Puoti, Peter Hunziker, Chantal Wicky, Fritz Müller

**Affiliations:** 1 Department of Biology, University of Fribourg, Fribourg, Switzerland; 2 Functional Genomics Center Zürich, University/ETH Zurich, Zürich, Switzerland; Texas A&M University, United States of America

## Abstract

Biochemical purifications from mammalian cells and Xenopus oocytes revealed that vertebrate Mi-2 proteins reside in multisubunit NuRD (Nucleosome Remodeling and Deacetylase) complexes. Since all NuRD subunits are highly conserved in the genomes of *C. elegans* and *Drosophila*, it was suggested that NuRD complexes also exist in invertebrates. Recently, a novel dMec complex, composed of dMi-2 and dMEP-1 was identified in *Drosophila*. The genome of *C. elegans* encodes two highly homologous Mi-2 orthologues, LET-418 and CHD-3. Here we demonstrate that these proteins define at least three different protein complexes, two distinct NuRD complexes and one MEC complex. The two canonical NuRD complexes share the same core subunits HDA-1/HDAC, LIN-53/RbAp and LIN-40/MTA, but differ in their Mi-2 orthologues LET-418 or CHD-3. LET-418 but not CHD-3, interacts with the Krüppel-like protein MEP-1 in a distinct complex, the MEC complex. Based on microarrays analyses, we propose that MEC constitutes an important LET-418 containing regulatory complex during *C. elegans* embryonic and early larval development. It is required for the repression of germline potential in somatic cells and acts when blastomeres are still dividing and differentiating. The two NuRD complexes may not be important for the early development, but may act later during postembryonic development. Altogether, our data suggest a considerable complexity in the composition, the developmental function and the tissue-specificity of the different *C. elegans* Mi-2 complexes.

## Introduction

Epigenetics encompasses all inheritable changes capable of modulating gene expression that are not encoded by the DNA sequence itself. Such changes include modifications at the chromatin level, which can be achieved by four processes: DNA methylation, histone modifications, ATP-dependent chromatin remodeling and histone variant incorporation. The study of chromatin remodeling complexes has revealed a surprising complexity in the composition and the function of such complexes (reviewed in [1]). Based on sequence and structure, the ATPase subunits of these complexes are divided into four families: the SWI/SNF, ISWI, INO80 and CHD families. The CHD family is characterized by chromodomain containing proteins, including the Mi-2 proteins.

Mi-2 was first identified as an autoantigen in patients with dermatomyositis [2], [3], and as a key component of the Nucleosome Remodeling and histone Deacetylase (NuRD, also called NURD or NRD) complex (reviewed in [4], [5]). The vertebrate Mi-2/NuRD complex contains at least seven polypeptides. In addition to the Mi-2 protein, it also includes the class I histone deacetylases HDAC1 and HDAC2, the histone-binding proteins RbAp46/48, the methyl-binding MBD proteins and the metastasis-associated MTA proteins (reviewed in [5]). There is conflicting evidence regarding the exact composition of the NuRD complex because the vertebrate genome encodes at least two homologues for most of the NuRD subunits, including the two Mi-2 isoforms Mi-2α and Mi-2β [5]. The existence of such isoforms suggests that the vertebrate NuRD complex might not be a single molecular species and that the subunit heterogeneity reflects a functional specialization (reviewed in [5]).

In *Drosophila*, the existence of a NuRD complex has been strongly suggested by several interaction studies [6], [7], [8], [9], [10]. Recently, a new containing dMi-2 protein complex, dMec, was characterized in *Drosophila*. dMec is composed of dMi-2 and dMEP-1 [11] and is clearly distinct from the NuRD complex. dMec, which constitutes the major dMi-2 containing complex in *Drosophila* cells, is strongly expressed in embryos but its role during embryogenesis is not known. It is also involved in the repression of proneural genes of the achaete-scute complex [11].

The *C. elegans* genome encodes two Mi-2 homologues, LET-418 and CHD-3 [12]. Despite a high degree of similarity between the two proteins, the mutant phenotype resulting from loss of function of the respective genes is quite different. Strong loss-of-function alleles of *let-418* lead to sterility, a protruding vulva, ectopic induction of the vulva precursor cell P8.p and, in the absence of maternal contribution, arrest of larval development at the mid-L1 stage associated with an ectopic expression of the P granule component PGL-1 in somatic cells [12]. P granules are large, non-membrane bound, ribonucleoprotein organelles found in the germline cytoplasm of most, if not all animals [13]. This suggested that the activity of *let-418* is required to repress germline specific genes in somatic cells during development [14]. Furthermore, *let-418* negatively regulates the expression of the Hox gene *lin-39* [14]. In contrast to *let-418* animals, *chd-3* mutants show no obvious phenotype. A role for *chd-3*, however, becomes visible in *let-418;chd-3* double mutants, which arrest as embryos in the absence of a maternal *let-418* contribution [12]. Both, *let-418* and *chd-3* play a role during vulva formation. In the wild-type hermaphrodite, the vulva is formed from the descendants of three of six equivalent vulval precursor cells (VPCs). The three cells are induced by multiple cell signaling pathways to adopt vulval cell fates. A large group of genes, the synthetic multivulva (SynMuv) genes, act redundantly to repress vulval differentiation. The SynMuv genes fall into two subgroups, termed A and B. While a single loss-of-function mutation in each subgroup does not result in an obvious vulval induction defect, a mutation in each of the two classes gives rise to a robust Muv phenotype (for a review see [15]). We found that *let-418* is a class B synMuv gene, whereas *chd-3* does not show a synMuv phenotype [12]. However, *chd-3* is required redundantly with *let-418* for the proper execution of the 2° cell fate in vulval precursor cells and plays a role in the specification of the pharyngeal precursor cells [12].

To gain further insight into the different functions of the two Mi-2 paralogues in *C. elegans*, we have characterized the LET-418 and CHD-3 containing complexes. Here we show that *C. elegans* harbours two distinct and previously undescribed Mi-2/NuRD complexes and an additional LET-418 containing complex, the MEC complex. The latter also contains the Krüppel-like protein MEP-1 and represents the major LET-418 containing regulatory complex during *C. elegans* embryonic and early larval development. Our data suggest that it acts before the bean stage to restrict the germline potential of somatic cells.

## Results

### LET-418 and CHD-3 are members of two distinct NuRD complexes

**Figure 1:**
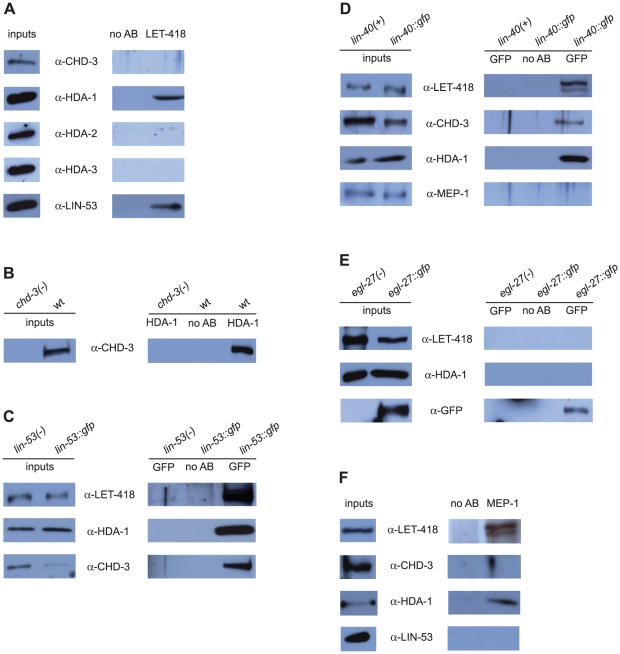
Two NuRD complexes and a MEC complex are present in *C. elegans*. (A) LET-418 interacts with HDA-1 and LIN-53, but not with CHD-3 nor with HDA-2 and HDA-3. Extracts from wild-type mixed-stage worms were precipitated with either α-LET-418 or no antibodies (negative control). Inputs and immunoprecipitates were subjected to Western analysis and immunoblotted with antibodies directed against proteins indicated next to each panel. (B) HDA-1 binds to CHD-3. Extracts from wild-type or *chd-3(eh4)* mixed-stage worms were precipitated with either α-HDA-1 or no antibodies (negative control). Inputs and immunoprecipitates were subjected to Western analysis and immunoblotted with antibodies directed against CHD-3. (C) LIN-53::GFP interacts with LET-418, HDA-1 and CHD-3. Extracts from *lin-53::gfp* or *lin-53(-)* mixed-stage worms were precipitated with either α-GFP or no antibodies (negative control). Inputs and immunoprecipitates were subjected to Western analysis and immunoblotted with antibodies directed against proteins indicated next to each panel. (D) LIN-40::GFP interacts with LET-418, HDA-1 and CHD-3, but not with MEP-1. Extracts from *lin-40::gfp* or *lin-40(+)* mixed-stage worms were precipitated with either α-GFP or no antibodies (negative control). Inputs and immunoprecipitates were subjected to Western analysis and immunoblotted with antibodies directed against proteins indicated next to each panel. (E) EGL-27::GFP does not interact with LET-418 nor with HDA-1. Extracts from *egl-27::gfp* or *egl-27(-)* mixed-stage worms were precipitated with either α-GFP or no antibodies (negative control). Inputs and immunoprecipitates were subjected to Western analysis and immunoblotted with antibodies directed against proteins indicated next to each panel. The membrane was reprobed with anti-GFP as a positive control. (F) MEP-1 interacts with LET-418 and HDA-1, but not CHD-3 nor with LIN-53. Extracts from wild-type mixed-stage worms were precipitated with either α-MEP-1 or no antibodies (negative control). Inputs and immunoprecipitates were subjected to Western analysis and immunoblotted with antibodies directed against proteins indicated next to each panel. Input: 5% of the immunoprecipitate; IP: 100% of the immunoprecipitate (1 mg of proteins). All co-immunoprecipitation experiments were reproducibly performed at least twice.

Because the putative null alleles *let-418(s1617)* and *chd-3(eh4)* have a different phenotype we expected the two proteins LET-418 and CHD-3 to reside in separate complexes. As predicted, we found that anti-LET-418 antibodies failed to co-precipitate CHD-3 (Figure 1A). As a positive control we used anti-HDA-1 antibodies, since LET-418 interacts with HDA-1/HDAC (Figure 1A, second lane). These results show that LET-418 and CHD-3 do not co-precipitate, suggesting that they reside in different complexes.

Next we searched for interactions between LET-418 or CHD-3 with various orthologues of the core NuRD components encoded by the *C. elegans* genome. First we tested whether LET-418 interacts with the three class I histone deacetylases, HDA-1, HDA-2 and HDA-3 [16]. However, LET-418 interacted only with HDA-1, but not with the two other HDACs HDA-2 or HDA-3 (Figure 1A). Similarly, we found that CHD-3 co-immunoprecipitated with HDA-1 (Figure 1B).

RbAp46 and RbAp48 (pRB-associated proteins p46 and p48, also known as RBBP7 and RBBP4, respectively) are two other members of the mammalian NuRD complex (reviewed in [17]). The *C. elegans* genome encodes two RbAp homologues, LIN-53/RBA-2 and RBA-1. To test a possible interaction of LIN-53/RbAp with the two *C. elegans* Mi-2 orthologues, a protein extract was prepared from worms carrying a partially rescuing *lin-53::gfp* fusion construct [18]. Anti-GFP antibodies against LIN-53::GFP co-immunoprecipitated both LET-418, CHD-3 and HDA-1 (Figure 1C). In a parallel approach we could also co-precipitate LIN-53 with specific anti-LET-418 antibodies (Figure 1A). Moreover, purification of LIN-53::TAP from a strain carrying a rescuing *lin-53::tap* construct yielded the same results (data not shown and see below). We also tested the second *C. elegans* RbAp46/48 orthologue RBA-1 using a strain expressing a RBA-1::TAP fusion protein. However, we found that RBA-1::TAP interacted neither with LET-418, nor with CHD-3 or HDA-1 (data not shown). Thus, our data suggested that the *C. elegans* Mi-2 LET-418 and CHD-3 interact only with LIN-53/RbAp, but not with RBA-1/RbAp.

The MTA proteins are additional components of the vertebrate NuRD complexes (reviewed in [5]). The genome of *C. elegans* encodes two proteins with homology to MTA1, namely LIN-40 (also called EGR-1) and EGL-27. LIN-40 is structurally more related to the vertebrate MTA family than EGL-27 [5]. Using protein extracts made from worms carrying a rescuing *lin-40::gfp* fusion construct [19], we could co-immunoprecipitate LET-418, CHD-3 and HDA-1 with LIN-40::GFP (Figure 1D). We also tested the second MTA orthologue EGL-27. Immunoprecipitation of a rescuing EGL-27::GFP construct [20] yielded neither LET-418, nor CHD-3 or HDA-1 (Figure 1E and data not shown). Altogether, these results suggested that LET-418, CHD-3 and HDA-1 interact with LIN-40/MTA but not with EGL-27/MTA.

To corroborate our co-immunoprecipitation data, we undertook a complex purification using the TAP (tandem affinity purification) method that was adapted to *C. elegans*. Since we could not obtain a full-length LET-418 tagged protein, we tagged the LIN-53/RbAp protein that interacts with both *C. elegans* Mi-2 orthologues (see above). The resulting *lin-53::tap* fusion construct was able to rescue the synMuv phenotype of *lin-15A;lin-53(n833)* worms. Upon tandem affinity purification of LIN-53::TAP, we co-precipitated the NuRD subunits LET-418/Mi-2, CHD-3/Mi-2, HDA-1/HDAC and LIN-40/MTA, but not the orthologues EGL-27/MTA and RBA-1/RbAp (Table 1). Besides the NuRD components, we isolated additional proteins, among them several members of the DRM and the NURF-like complexes. This was expected since these two protein complexes also contain LIN-53 [21], [22].

**Table 1:** LIN-53::TAP co-precipitates NuRD complex core components.

Protein	Description
LIN-53	Nucleosome remodeling factor
LET-418	Mi-2 orthologue
CHD-3	Mi-2 orthologue
HDA-1	Histone deacetylase 1
LIN-40	MTA1 orthologue
NURF-1	Nucleosome remodeling factor
ISW-1	Chromatin remodeling complex ISWI
LIN-59	Putative transcription factor ASH1
LIN-9	Rb pathway protein
LIN-37	-
UNC-82	Predicted serine/threonine protein kinase
TAG-235	Histone acetyltransferase
CPSF-1	mRNA cleavage and polyadenylation factor

LIN-53::TAP containing complexes were subjected to tandem affinity purification. Proteins co-purified with LIN-53::TAP were identified by liquid chromatography tandem mass spectrometry. Proteins were identified using a ProteinLynx Global server and Mascot Search engines.

Altogether biochemical data suggests the presence of two different NuRD complexes in *C. elegans*. They differ in their Mi-2 orthologues LET-418 or CHD-3, but share the same core subunits HDA-1, LIN-53 and LIN-40. Their respective homologous proteins HDA-2/-3, RBA-1 and EGL-27 did not interact, although we cannot rule out that in some tissues or developmental stages they may reside in the same complex.

### The LET-418/Mi-2 containing NuRD complex functions in the synMuv B pathway

LET-418 acts in the SynMuv B pathway [12]. If LET-418 functions during vulva development as a component of a NuRD complex, we would expect that the genes encoding the other components of this complex also behave as synMuv B genes. Consistent with this hypothesis, *hda-1*, *lin-53* and *lin-40* are also synMuv B genes [12], [18], [23], [24], [25]. Depletion of *rba-1* and *egl-27* in a *lin-15A*, *lin-15B* or *lin-35* background, on the other hand, did not result in a synMuv phenotype (Table 2 and [26]), suggesting that they are not synMuv genes. Likewise, *chd-3*, *hda-2* and *hda-3* are also not part of the synMuv B nor the synMuv A pathways [12], [18]. Altogether, these data are of twofold interest. First, they support the idea that a LET-418, HDA-1, LIN-53 and LIN-40 containing NuRD complex may act via the synMuv B pathway. Secondly, they provide genetic evidence that CHD-3, HDA-2, HDA-3, RBA-1 and EGL-27 do not act in the NuRD complex with LET-418.

**Table 2:** *rba-1 and egl-27* are not synMuv B genes.

Genotype	% Muv (n)
N2; *rba-1(RNAi)*	0 (132)
*lin-15A(n767); rba-1(RNAi)*	0 (365)
*lin-15B(n744); rba-1(RNAi)*	0 (9)*
*lin-35(n745); rba-1(RNAi)*	0 (9)*
*lin-15A(n767); lin-53(RNAi)*	74 (243)
N2; *egl-27(RNAi)*	0 (798)
*lin-15A(n767); egl-27(RNAi)*	0 (899)
*lin-15B(n744); egl-27(RNAi)*	0 (9)*
*lin-35(n745); egl-27(RNAi)*	0 (40)
*lin-15A(n767); lin-40(RNAi)*	0 °
*egl-27(n170);* HT115	0 (172)
*egl-27(n170); lin-15A(RNAi)*	0 (253)
*egl-27(n170); lin15B(RNAi)*	0 (450)
*egl-27(n170); lin-35(RNAi)*	0 (395)
*lin-40(ku285); lin-15A(RNAi)*	22 (11)

*no adults or only few adults were obtained because most of the worms arrested as larvae.

° synMuv only with *lin-40(ku285)* allele (Chen *et al.*, 2001).

The genetic background, the percentage of synMuv and the number of animals counted (n) are indicated.

### MEP-1 and LET-418 reside in a complex distinct from NuRD

Previously, it was found that the *C. elegans* Krüppel-like protein MEP-1 interacts with LET-418 and HDA-1 ([27] and Figure 1F). To further characterize this interaction and to see whether it occurs in the context of a NuRD complex, we tested if MEP-1 binds to the NuRD components LIN-53/RbAp and LIN-40/MTA. However, we could not co-precipitate MEP-1 with LIN-53 (Figure 1F) nor with LIN-40::GFP (Figure 1D). We then asked whether MEP-1 was able to physically interact with the second *C. elegans* Mi-2 orthologue CHD-3. We found that CHD-3 did not co-precipitate with MEP-1 (Figure 1F). These results suggested that MEP-1 only interacts with LET-418 and HDA-1 but not with CHD-3 to form a complex that is distinct from the previously described NuRD complexes. By analogy to the *Drosophila* dMec, we named the *C. elegans* LET-418 and MEP-1 containing complex “MEC complex”. However, in the dMec no histone deacetylase activity was detected [11].

Both, arrested *let-418* and *mep-1* L1 larvae show ectopic expression of the P granule component PGL-1 in their somatic cells [27], suggesting that a LET-418 and MEP-1 containing MEC complex is required to repress *pgl-1* in somatic cells. To test whether other LET-418 interacting proteins may also be involved in the control of *pgl-1* expression, we fed L1 larvae with dsRNA corresponding to all genes encoding NuRD complex components. Upon staining with anti-PGL-1 antibodies, we found ectopic PGL-1 expression only in *let-418(RNAi)* and *mep-1(RNAi)*, but not in *lin-53*/RbAp, *lin-40*/MTA, *chd-3*/Mi-2, *hda-2*/HDAC, *hda-3*/HDAC, *rba-1*/RbAp and *egl-27*/MTA RNAi-depleted worms (Table 3). Due to the high percentage of dead embryos in *hda-1(RNAi)* worms [16], we could not analyze the effect of HDA-1 on PGL-1 expression.

**Table 3:** The MEC complex regulates *pgl-1* expression in L1 larvae.

	Gene inhibited by RNAi a	ectopic PGL-1 staining	*pgl-1* mRNA fold change
	control RNAi	no	1
MEC complex	*let-418*	yes^b^	54.2
	*mep-1*	yes^b^	62.7
	*hda-1*	n.d.^d^	n.d.^d^
LET-418 NuRD complex	*let-418*	yes^b^	54.2
	*hda-1*	n.d.^d^	n.d.^c^
	*lin-53*	no^c^	0.5
	*lin-40*	no	0.5
CHD-3 NuRD complex	*chd-3*	no	0.5
	*hda-1*	n.d.^d^	n.d.^d^
	*lin-53*	no^b^	0.5
	*lin-40*	no	0.5
non NuRD homologues	*hda-2*	no	0.03
	*hda-3*	no	0.1
	*rba-1*	no	1.8
	*egl-27*	no	0.5

a Efficiency of all dsRNAs were tested by looking for the expected phenotype.

b Unhavaithaya *et al.* (2002).

c Wang *et al.* (2005).

d could not be determined due to the very high percentage of dead embryos in *hda-1(RNAi)* worms.

The genetic background and the presence/absence of ectopic P granules in the different mutant background are indicated, as well as the fold change of *pgl-1* mRNA.

In a parallel approach, we performed qRT-PCR experiments to determine the *pgl-1* mRNA levels in animals depleted for *let-418*, *mep-1* and all genes encoding NuRD complex components. Consistent with the previous results, we found that *pgl-1* mRNA levels were upregulated only in *let-418(RNAi)* and *mep-1(RNAi)* L1 larvae, but not in animals that were RNAi-depleted for *lin-53*, *lin-40*, *chd-3*, *hda-2*, *hda-3*, *rba-1* and *egl-27* (Table 3). Altogether, the genetic data demonstrated that *pgl-1* is jointly regulated by LET-418 and MEP-1, but not by NuRD subunits, thus supporting the notion that MEC and NuRD are distinct functional complexes.

### The MEC and NuRD complexes differentially repress *lag-2::gfp* expression

**Figure 2:**
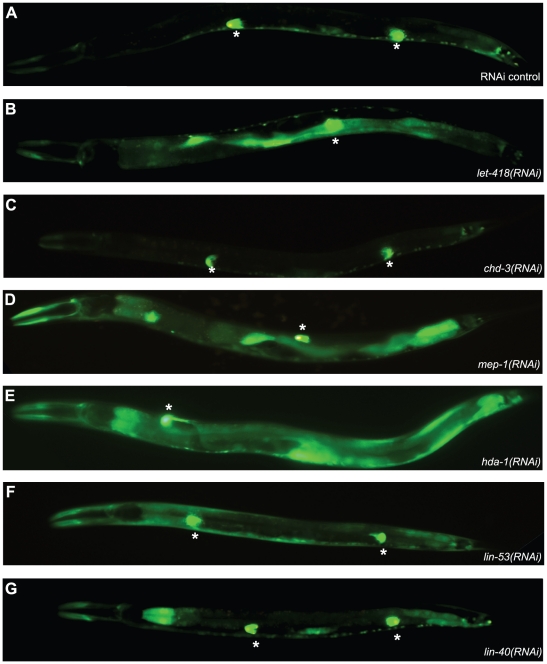
The MEC complex negatively regulates *lag-2::gfp* expression in the gut. The *lag-2::gfp* transgene is expressed in the gut of *let-418(RNAi)*, *mep-1(RNAi)* and *hda-1(RNAi)* L3 larvae. (A) L3 worms carrying *lag-2::gfp* transgene and fed bacteria containing empty vector (RNAi control) (A) show expression in the Distal Tip Cells (DTC) and in the ventral nerve cord. No ectopic expression is observed. (B, D–E) *lag-2::gfp* is ectopically expressed in the gut of *let-418(RNAi)* (B), *mep-1(RNAi)* (D) and *hda-1(RNAi)* (E) L3 larvae. (C) No ectopic expression is observed in *chd-3(RNAi)* L3 larvae. (F) *lag-2::gfp* is mainly expressed in the epidermis in *lin-53(RNAi)* L3 larvae. (G) *lag-2::gfp* is ectopically expressed only in two cells in the most anterior part of the intestine in *lin-40(RNAi)* L3 larvae. The asterisk marks the DTC.

Earlier, it was shown that LET-418 negatively regulates the expression of a *lag-2::gfp* reporter gene in the gut [28]. In wild-type animals *lag-2::gfp* is expressed in the Distal Tip Cells (DTCs) and weakly along the ventral nerve cord (Figure 2A and [29]), whereas in *let-418* mutants it shows an additional strong expression in the intestine (Figure 2B and [28]). A wild-type expression pattern was also observed in *chd-3(RNAi)* animals (Figure 2C), suggesting that CHD-3 is not involved in the regulation of *lag-2::gfp*. To determine, which of the LET-418 containing complexes might be responsible for the suppression of the ectopic expression, we analyzed the *lag-2::gfp* expression in L3 larvae that were RNAi-depleted for *mep-1*, *hda-1*, *lin-53* and *lin-40*. To our surprise, we reproducibly found three characteristic types of *lag-2::gfp* expression patterns. Strong intestinal *lag-2::gfp* expression was only observed in *let-418(RNAi)*, *mep-1(RNAi)* and *hda-1(RNAi)* worms (Figures 2B, 2D–2E) and [30]), whereas in *lin-53(RNAi) lag-2::gfp* was expressed mainly in the epidermis but clearly not in the intestine (Figure 2F). Finally, in *lin-40(RNAi)* worms, *lag-2::gfp* was strongly expressed only in the two most anterior cells of the intestine and in a weaker manner in a few posterior intestinal cells (Figure 2G). Worms that were RNAi-depleted for the non-NuRD proteins encoding genes *hda-2*, *hda-3*, *rba-1*, *egl-27* and *mbd-2*, had a wild-type *lag-2::gfp* expression pattern (data not shown). These results suggested that the MEC complex might negatively regulate *lag-2::gfp* expression in the intestine. *lin-53(RNAi)* and *lin-40(RNAi)* produced different *lag-2::gfp* expression patterns (see Figure 2F–2G), suggesting that NuRD and/or other LET-418 containing complexes may play additional, tissue-specific roles in the regulation of the *lag-2::gfp* reporter outside of the gut.

### LET-418 and MEP-1 regulate common target genes

The repression of the germline gene *pgl-1* and the transgene *lag-2::gfp* in the gut by LET-418 and MEP-1 implies a common role for the two proteins in regulating tissue-specific gene expression throughout development. To identify potential target genes, we performed a genome wide gene profile analysis in *let-418* and *mep-1* depleted animals. For our experiments we chose arrested L1 larvae, which also show ectopic P granule expression [12]. Since *mep-1(q660)* mutants are sterile [31], we used RNA interference to generate *mep-1* and *let-418* depleted worms. Animals fed with bacteria expressing *gfp* dsRNA (pPE128.110 in HT115) were used as reference sample. Deregulated genes with a *p*-value of ≤0.01 and fold change ≥ ±2 were further analyzed. The microarray results were validated by qRT-PCRs on ten randomly selected genes deregulated in both *let-418(RNAi)* and *mep-1(RNAi)* (five up- and five downregulated genes). The results confirmed the microarray data for all ten genes (Figure S1).

**Figure 3:**
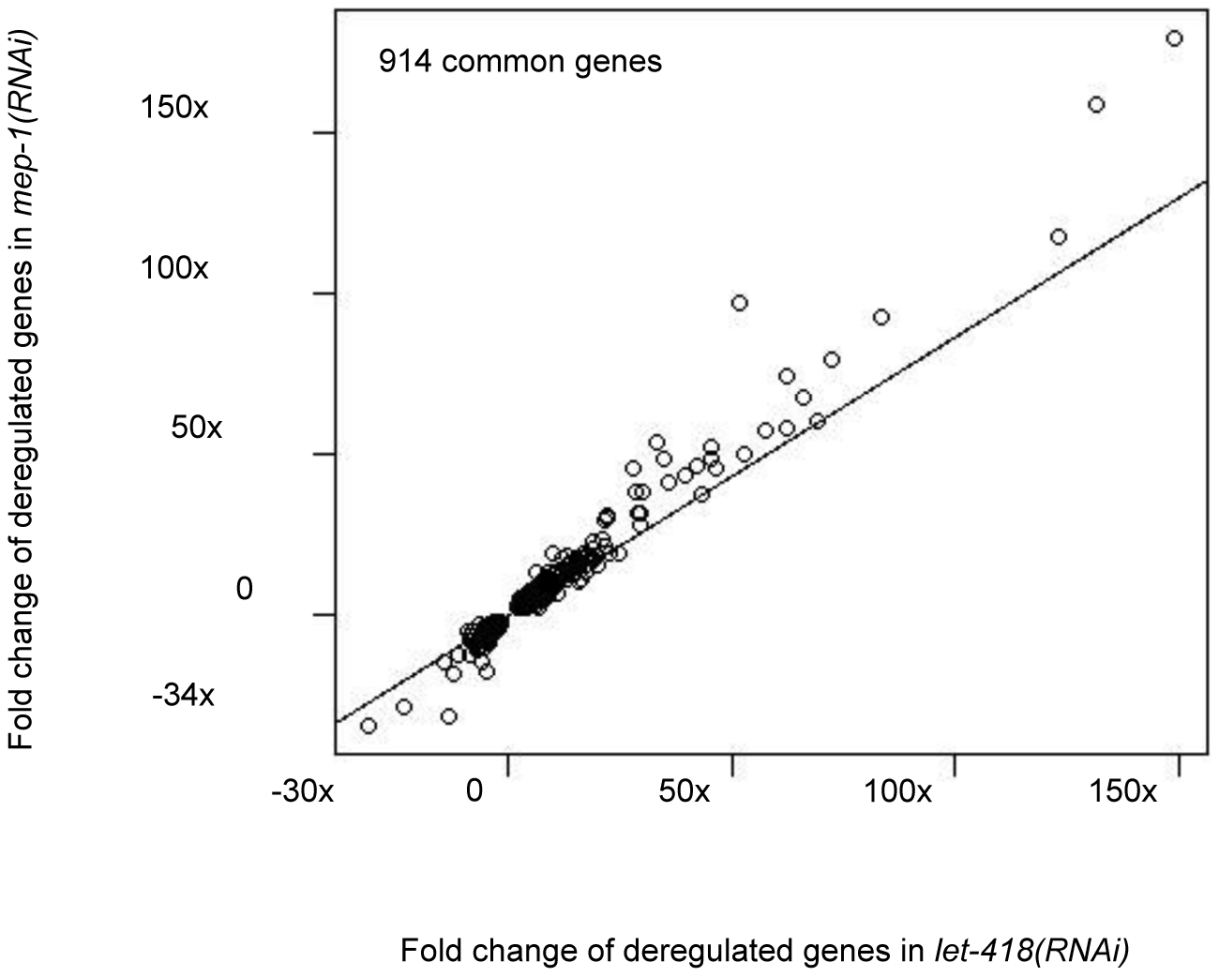
LET-418 and MEP-1 regulate common target genes. A strong correlation is observed between the genes deregulated in *let-418(RNAi)* and *mep-1(RNAi)* L1 larvae. The fold change of each of the common genes was plotted on the graph (X-axis: fold change of *let-418(RNAi)* genes, Y-axis: fold change of the *mep-1(RNAi)*). Each circle represents a single gene. A standard correlation factor of R = 0.98 was calculated according to the linear regression.

A total of 1113 genes showed changed expression levels in *let-418(RNAi)* L1, whereas 1104 genes were deregulated in *mep-1(RNAi)* worms. Given the similar phenotype of *let-418(RNAi)* and *mep-1(RNAi)* animals and the physical interaction of LET-418 and MEP-1, we expected a comparable gene expression profile. Indeed, we found that 914 (82%) of the deregulated genes were common between *let-418(RNAi)* and *mep-1(RNAi)* animals (henceforth referred to as “common genes”). The majority of them (70%) were upregulated. Analyses using the statistical software MAGMA with R-scripts revealed a very strong correlation between their deregulation pattern of these genes. A standard correlation factor of R = 0.98 was calculated according to the linear regression (R = 1 means that the relation is linear), demonstrating that gene expression was deregulated very similarly in *let-418(RNAi)* and *mep-1(RNAi)* depleted L1 larvae (Figure 3). The high degree of correlation between the expression profiles is consistent with the idea that most, if not all, of those genes are controlled by the same MEC complex.

To test whether LET-418 and MEP-1 can also function independently from each other at this stage of development, we focused on the 18% of genes that were deregulated exclusively on either the *let-418(RNAi)* or the *mep-1(RNAi)* microarray. The expression of most of these genes was only moderately affected with a fold change around ±2. We tested the expression levels of 12 randomly selected genes by qRT-PCR analysis (six specifically deregulated genes were chosen on each microarray list). We found that the expression of six of them was not affected, neither in *let-418(RNAi)* nor in *mep-1(RNAi)* animals, whereas the six remaining genes were deregulated in both, *let-418(RNAi)* and in *mep-1(RNAi)* L1 larvae (Figure S2A–S2B). Thus, the 12 tested genes corresponded either to false negative or false positive signals on their microarrays and we could find no evidence for LET-418 and MEP-1 acting independently from each other. Therefore, we can conclude that MEC represents the major LET-418/Mi-2 containing gene regulatory complex acting during early larval development in *C. elegans*.

### LET-418 and MEP-1 regulate germline specific and early embryonic genes

Since *let-418* and *mep-1* were proposed to repress the germline potential in somatic cells during embryonic and early larval development [27] and our own observation), we were interested to determine how many germline expressed genes were deregulated in *let-418(RNAi)* and *mep-1(RNAi)* depleted L1 larvae. Recently, Wang et al. [32] have identified 4699 germline expressed genes from a SAGE library constructed from dissected *C. elegans* hermaphrodite gonads from young adults. They correspond to about 21% of all predicted *C. elegans* genes, and henceforth will be referred to as “germline genes”. We compared them with our 914 common genes co-regulated by LET-418 and MEP-1 and identified 222 (24.3%) putative germline genes among them. Although the germline genes were statistically not overrepresented among the deregulated genes, the fact that most of them (187 genes) were upregulated indicated a possible shift of the gene expression pattern towards germline specificity in *let-418(RNAi)* and *mep-1(RNAi)* depleted L1 larvae,

To further investigate this issue, we have searched for genes which expression is enriched in, or specific for germ cells. Such genes are suggested to have roles specific to germline functions. Since in *C. elegans* L1 larvae germline proliferation has not yet begun, the larvae only contain two germ cells (Z2 and Z3). Therefore, we expected germline-specific genes to be underrepresented or even absent from L1 larvae. Among the 4699 germline genes, Wang et al. identified 733 (15.6%) germline-enriched and 330 (7%) germline-specific genes. We have compared their data with the list of our common genes and found that they include 19 (8.5%) germline-enriched and 40 (18%) germline-specific genes. With the exception of two germline-enriched and one germline-specific genes they are all overexpressed and, particularly, the germline-specific genes are statistically overrepresented among the common genes. The upregulation of germline-enriched and germline-specific genes suggests that they may be ectopically expressed in the soma, an assumption that is also supported by the finding of P granules in intestinal and some hypodermal cells of *let-418(RNAi)* and *mep-1(RNAi)* depleted L1 animals [27]. We found that 17 of the currently 41 known genes encoding *C. elegans* P granule components [13] are derepressed in *let-418(RNAi)* and *mep-1(RNAi)* depleted L1 larvae, including *gld-1*, *gld-3*, *glh-1* and *glh-2*, *pgl-1*, *pos-1*, *deps-1* and others. Further examples of upregulated germline-specific genes are *him-3*, *htp-1*, *htp-2* and *htp-3*, which encode meiosis-specific HORMA domain-containing proteins involved in synaptonemal complex formation and meiotic chromosome segregation [33], [34]. Altogether, our expression data support the idea that *let-418* and *mep-1* are involved in the repression of the germline potential of somatic cells in L1 larvae.

**Figure 4:**
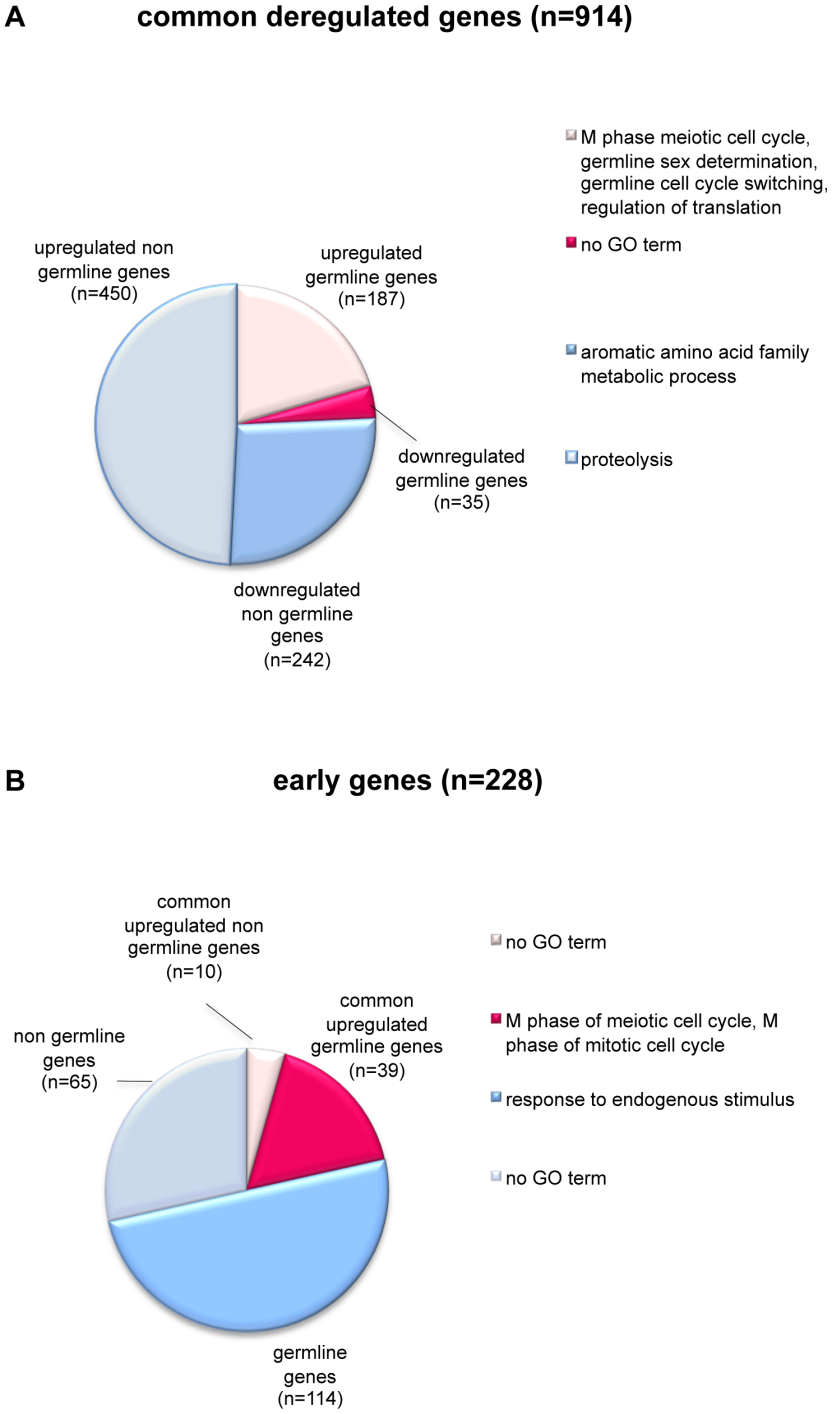
LET-418 and MEP-1 regulate germline and early embryonic genes. Pie charts show repartition and functional annotation of the common deregulated genes (A) and the early genes (B) according to gene ontology (GO) annotations. The GO terms are indicated on the right of each pie chart. (A) The pie chart shows the repartition of the 914 common deregulated genes compared to the list of 4699 germline genes. The common genes are divided into four groups: upregulated germline (rose slice) and non germline (gray slice) genes; and downregulated germline (pink slice) and non germline (blue slice) genes. (B) The pie chart shows the repartition of the 228 early genes compared to the common upregulated genes and to the list of 4699 germline genes. 49 common upregulated genes are also early genes. They are subdivided in germline (pink slice) and non germline (rose slice) genes. The remaining early genes, that are not targets of MEC, are subdivided in germline (blue slice) and non germline (gray slice) genes.

To gain further insight into the biological roles of the common genes, we analyzed the gene ontology (GO) terms associated with them by using the FatiGO+ software [35]. We found that the 187 upregulated germline genes are implicated in biological processes associated to hermaphrodite germline sex determination, M phase of meiotic cell cycle, germline cell cycle switching (the switch from mitotic to meiotic cell cycle) and regulation of translation (Figure 4A). They also comprise various genes encoding P granule components, such as *gld-1* and *gld-3*, *glh-1* and *glh-2*, *pgl-1*, *pos-1* and *deps-1*. The remaining 450 upregulated common genes, that could not be classified among the germline genes, were annotated as genes involved in proteolysis (Figure 4A). The 242 downregulated non germline genes, finally, are implicated mainly in aromatic amino acid family metabolic processes. No GO terms could be associated with the 35 downregulated germline genes.

Among the common deregulated genes we found also 49 early embryonic genes (Figure 4B). They belong to a group of 228 genes, which are expressed at the beginning of embryogenesis and then downregulated to background levels by the onset of gastrulation [36]. Their expression temporally correlates with the developmental plasticity observed in *C. elegans* embryonic blastomeres, which is lost during gastrulation [36]. Among the 49 genes, 39 belong to the class of germline genes and are significantly enriched in the GO terms associated with M phase of meiotic cell cycle and M phase of mitotic cell cycle (Figure 4B). Interestingly, the 114 remaining germline genes among the early genes which downregulation in L1 larvae does not depend on the MEC complex, are associated with response to endogenous stimulus. The 65 early genes that do not belong to germline genes did not show any significant GO term (Figure 4B). Altogether these data suggested that LET-418 and MEP-1 are specifically required during early embryogenesis to downregulate the early genes with mitotic and meiotic functions.

Ectopic P granules in *mep-1(RNAi)* animals can first be seen at or shortly after the two-fold stage of embryogenesis ([27] and own observations). This suggested that *mep-1* and *let-418* must act prior to this stage to ensure normal development. To further identify the time laps in which *let-418* activity is required during early development, we shifted *let-418 temperature-sensitive* embryos at different embryonic stages from the permissive temperature (15°C) to the restrictive (25°C) temperature and followed their development. Embryos shifted to 25°C prior to the lima bean stage arrested at the L1 stage (Table 4). By contrast, embryos shifted to 25°C after the lima bean stage did not arrest and developed beyond L1 into mixed-stage larvae and fertile adults. Taken together, our results indicated that, in order to bypass the L1 larval arrest, the *let-418* activity is required prior to the lima bean stage, which corresponds to the time point when embryonic cell proliferation largely ceases and morphogenesis starts [37].

**Table 4:** *let-418* activity is required prior to the bean stage to prevent the L1 larval arrest.

	1–8 cells (17)	8–24 cells (20)	24–50 cells (19)	50- bean stage (21)	bean stage (23)	comma stage (23)	1.5x-stage (20)	2x-stage (20)	3x-stage (21)	newly hatched (30)
> L1	0%	0%	0%	0%	0%	90%	100%	100%	100%	100%
L1	100%	100%	100%	100%	100%	10%	0%	0%	0%	0%

LET-418 activity is required prior the bean stage to allow normal L1-L2 transition. *let-418(n3536)ts* embryos grown at 15°C were transferred at 25°C. Animals were scored according to their body length and morphological structures three days after hatching at 25°C. In parentheses: number of scored animals.

### LET-418 and CHD-3 have different functions and expression patterns

We were also interested to learn more about the function of *chd-3*, the second *C. elegans* Mi-2 orthologue. Therefore, we analyzed the gene expression profile of *chd-3* L1 larvae by using transcriptional microarrays. At this stage, *chd-3* animals have no obvious phenotype. Consistently, we found that the expression of only a few genes was affected. For that reason we set the minimal median fold change limit at 1.5 instead of 2 (with a *p*-value ≤0.01). After validation of the candidates by qRT-PCR, we ended up with only 9 deregulated genes; the expression of 7 of them was moderately downregulated whereas the remaining two target genes were upregulated (data not shown). No GO term could be attributed to this limited number of genes. Only one upregulated gene, *cyp-14A5*, was also deregulated in *let-418(RNAi)* and *mep-1(RNAi)* animals, however it was downregulated in the latter. Obviously, *chd-3* does not play much of a regulatory role at this stage.

**Figure 5:**
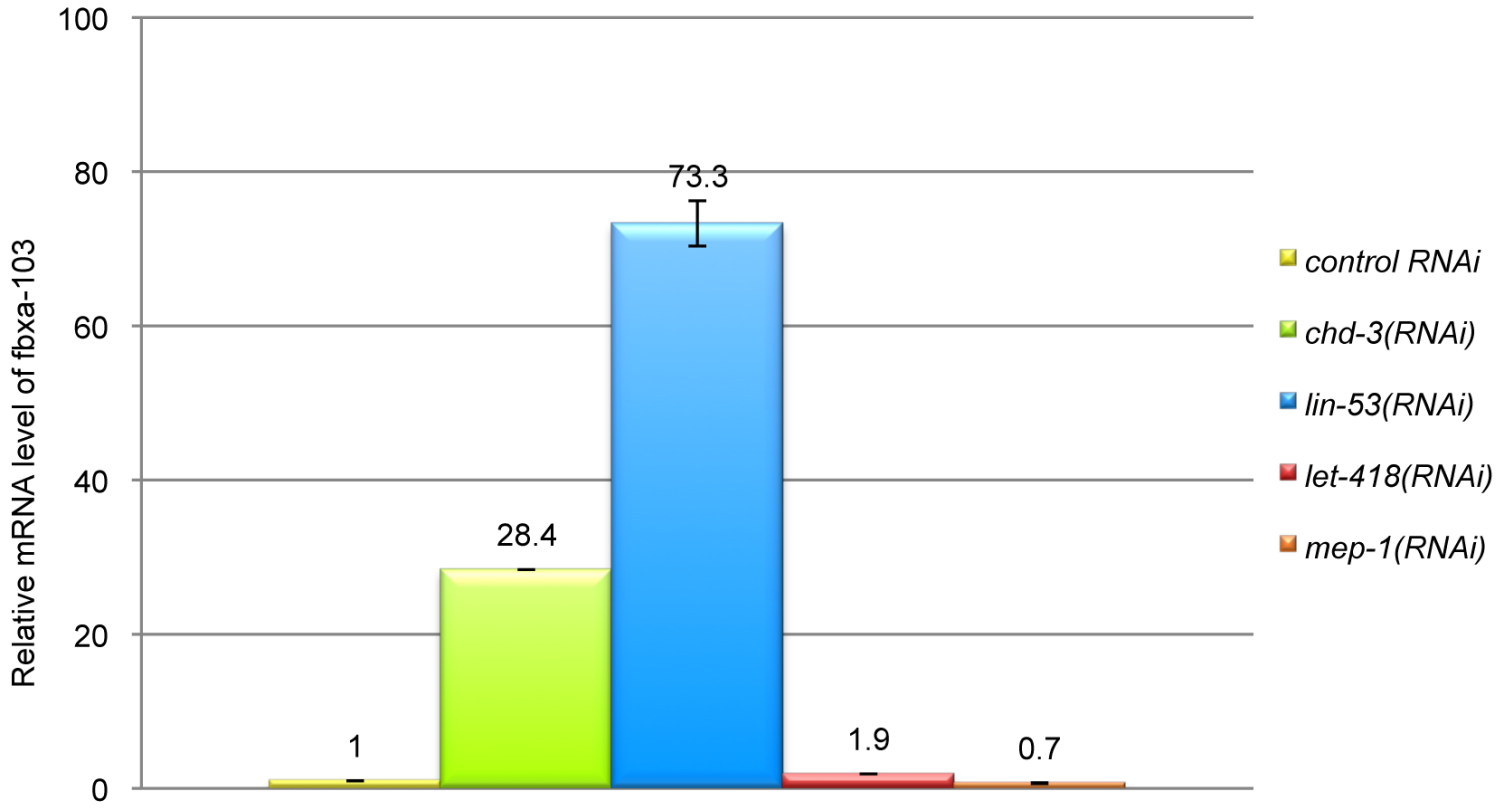
CHD-3 acts as a component of the NuRD complex to regulate *fbxa-103*. Fold change of *fbxa-103* mRNA was analyzed by qRT-PCR in diverse RNAi-treated L1 larvae, as indicated on the right of the panel. *fbxa-103* is upregulated in *chd-3(RNAi)* and *lin-53(RNAi)* but not in *let-418(RNAi)* nor in *mep-1(RNAi)* L1 larvae.

To test whether CHD-3 may act as a component of a NuRD complex, we analyzed the expression level of one of its target genes, *fbxa-103*, in worms RNAi-depleted for diverse NuRD members. *fbxa-103* encodes a protein with an F-box motif predicted to mediate protein-protein interactions. We found that *fbxa-*103 was upregulated in *chd-3(RNAi)* and *lin-53(RNAi)* animals, but not in *let-418(RNAi)* nor in *mep-1(RNAi)* worms (Figure 5). These data are consistent with the idea that CHD-3 negatively regulates the expression of *fbxa-103* in the context of a NuRD complex.

**Figure 6:**
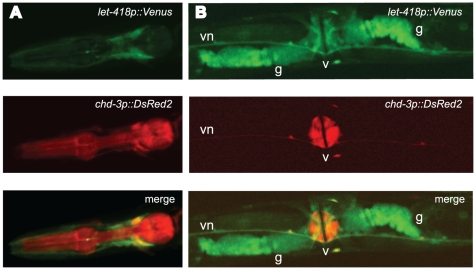
*let-418* and *chd-3* transcriptional reporters have different expression patterns. (A-B) Young adults animals carrying both a transcriptional *let-418p::Venus* (first lane) and a transcriptional *chd-3p::DsRed2* (second lane) reporter genes were analyzed by confocal microscopy. The third lane is the merged picture from both *let-418p::Venus* and *chd-3p::DsRed2* reporters. Expression is shown (A) in the head and (B) in the vulva and somatic gonad. v: vulva (ventral view); g: somatic gonad; vn: ventral nerve cord.

Next we have analyzed the developmental expression patterns of *let-418* and *chd-3*. Previously it was shown that *let-418* transcripts are present in the germline and maternally delivered to the early embryos, whereas *chd-3* mRNA is absent from the germline and appears first around the 20 cell stage after the onset of zygotic transcription ([12] and data not shown). To compare the expression patterns of the two genes during later larval development and in adults, we constructed transcriptional reporter genes by fusing the *let-418* promoter region with the *Venus* fluorescent reporter and the *chd-3* promoter region with the *DsRed2* reporter. For each reporter construct, three independent transgenic lines were analyzed. Both reporters were strongly expressed in most if not all cells of the embryo (data not shown). In young adults, both the *let-418* and *chd-3* transgenes were primarily expressed in the head, the vulva, the tail, the ventral nerve cord and the distal tip cells (Figure 6A–B and data not shown). The strong co-expression of *let-418* and *chd-3* in the vulva is in agreement with the finding that both genes are redundantly required for the specification of the 2° fate of P.5p and P.7p [12]. The *let-418p::Venus* construct was expressed in cells surrounding the pharynx, whereas the *chd-3p::DsRed2* construct was mainly located in the pharynx itself (Figure 6A). The specific pharyngeal expression of *chd-3* is in agreement with the finding that *chd-3*, but not *let-418* nor *mep-1*, plays a role in the pharyngeal precursor cells specification [38]. Another interesting difference was found in the somatic gonad, where only the *let-418* reporter, but not the *chd-3* reporter was strongly expressed (Figure 6B). These data suggested that *let-418* and *chd-3* may have distinct tissue-specific functions during the postembryonic development and in adults.

## Discussion

**Figure 7:**
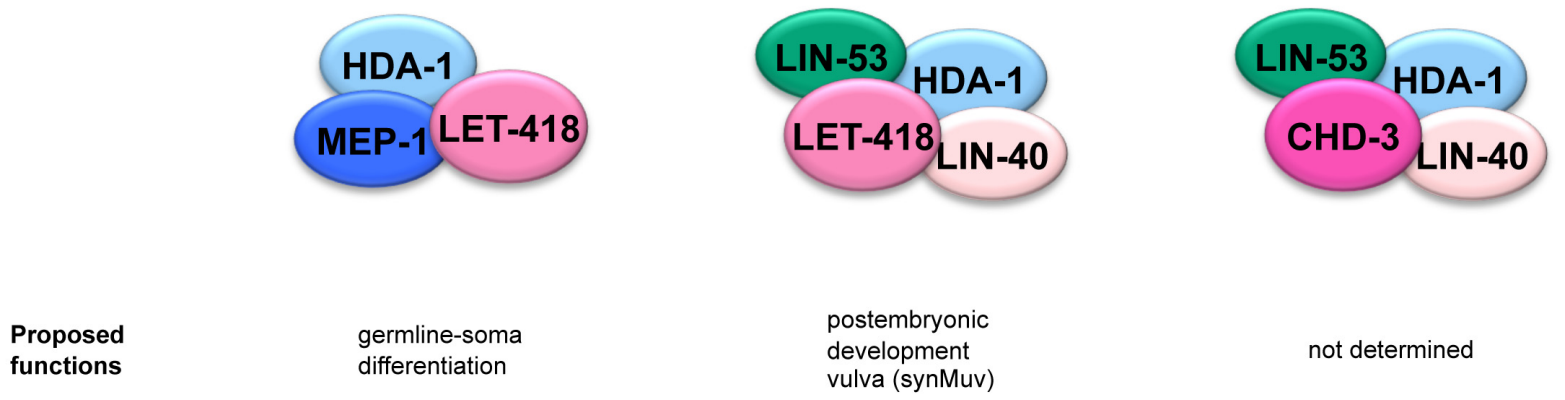
Summary figure of the complexes and their proposed roles. The two *C. elegans* Mi-2 proteins are members of at least three different complexes, two distinct NuRD complexes and a MEC complex.

This study brings an important contribution towards the understanding of the developmental roles of the *C. elegans* Mi-2 orthologues LET-418 and CHD-3. We demonstrate that the two *C. elegans* Mi-2 proteins are members of at least three different protein complexes, two distinct NuRD complexes and a MEC complex comprising LET-418/Mi-2, the Krüppel-like protein MEP-1 and the histone deacetylase HDA-1 (Figure 7). Our data suggest that MEC constitutes an important LET-418 containing gene regulatory complex throughout embryonic and early L1 development, whereas the two NuRD complexes rather function during later larval development, for instance, during vulval development.

Previous studies have revealed a physical interaction between LET-418, MEP-1 and HDA-1 ([27] and own data), suggesting that the three proteins reside in a common regulatory complex. Consistent with this idea, *let-418* and *mep-1* mutants share the same phenotype that includes an L1 larval arrest and derepression of germline specific genes in somatic cells of the arrested larvae. Furthermore, *mep-1(RNAi)* depleted *let-418* mutants also arrest at the L1 stage and show no additional synthetic phenotype, suggesting that the two proteins act together in the same biological process. Because of its embryonic lethal phenotype, we could not test whether depletion of *hda-1* causes a similar L1 larval phenotype, as would be expected if HDA-1 was also a member of this complex during early development. However, depletion of *let-418*, *mep-1* and *hda-1* resulted in an identically strong ectopic expression of a *lag-2::gfp* reporter gene in the intestine of L3 larvae. This suggested that a MEC complex containing LET-418, MEP-1 and HDA-1 is required for the negative control of a *lag-2::gfp* reporter in the gut during later larval development. This is in contrast to the situation in *Drosophila*, where dRPD3/HDAC did not physically associate with dMi-2 and dMEP-1 [11]. However, we cannot rule out that in *C. elegans* the composition of the MEC complex shows stage-specific differences. Moreover, the *C. elegans* MEC complex probably contains additional subunits, since fractionation of protein extracts from mixed-stage worms resulted in the co-elution of LET-418 and MEP-1 at an estimated complex size ranging from 0.8 to 1.5 MDa (Figure S3 and Materials and Methods S1).

Gene expression profiling experiments revealed that at least 82% of the deregulated genes in *let-418(RNAi)* and *mep-1(RNAi)* worms are tightly co-regulated, thus supporting the association of LET-418 and MEP-1 in a common regulatory MEC complex. Furthermore, our data demonstrate that MEC represents the major LET-418/Mi-2 containing gene regulatory complex during early *C. elegans* development. Most of the deregulated genes (about 70%) are upregulated in *let-418(RNAi)* and *mep-1(RNAi)* animals, suggesting that MEC mainly functions in transcriptional repression. Similarly, the *Drosophila* dMec complex was shown to contribute to the repression of proneural genes [11].

Based on the observation that P granule-like perinuclear structures are found in the somatic cells of arrested *let-418* and *mep-1* L1 larvae ([27] and own observations), it has been suggested that LET-418 and MEP-1 negatively regulate the germline potential of somatic blastomeres during early development. Consistently, we found that in arrested *let-418(RNAi)* and *mep-1(RNAi)* L1 larvae totally 187 germline genes were upregulated, among them 59 germline-enriched and germline-specific genes [32], [39]. The fact that only a fraction of the germline-specific genes encoded by the genome of *C. elegans* is derepressed in LET-418 and MEP-1 depleted animals suggest that the two proteins do not play a global role in the regulation of germline genes. Rather they affect specific transcripts, which are implicated in germline sex determination, M phase of meiotic cell cycle, germline cell cycle switching (the switch from mitotic to meiotic cell cycle) and regulation of translation. Among them are also 17 genes encoding components of P granules. Currently, it is not know why this particular subset of genes are controlled and why other germline-specific genes are not.

Most interestingly, *let-418* and *mep-1* control the transcription of specific early expressed embryonic genes, which are normally active at the beginning of embryogenesis and downregulated to background levels by the onset of gastrulation [36]. Their downregulation temporally correlates with the transition from a developmentally plastic state to the onset of differentiation [36], when embryonic cells become restricted in their cell fate potential and begin to acquire cell type identities. We found that LET-418 and MEP-1 are required for the downregulation of a subset of 44 early expressed genes that are specifically enriched in mitotic and meiotic functions. Thus, we may speculate that the MEC complex is already required in the early embryo to downregulate these embryonic genes in order to restrict the germline potential of early blastomeres once germline-soma separation has been achieved. LET-418 and MEP-1 may generally act during embryonic development before the onset of morphogenesis, when cells still divide. Consistent with this hypothesis, our temperature shift experiments indicated that the *let-418* activity must be present in the embryos before reaching the bean stage (i.e. when cell proliferation largely ceases and morphogenesis begins) in order to prevent L1 larval arrest. Furthermore, ectopic PGL-1 expression are first observed at the two-fold stage [27] and own data), suggesting that *mep-1* and *let-418* act before this stage to stably repress the germline potential of the somatic blastomeres.

Apart from an inappropriate expression of germline genes, somatic cells of arrested *let-418ts* L1 larvae seem to be correctly specified ([27] and own data). This is also pointed out by the observation that arrested *let-418ts* animals at 25°C, if shifted back to the permissive temperature of 15°C, can resume development and grow into fertile adults (unpublished data). Thus, LET-418 and MEP-1 are not required for the differentiation of the somatic blastomeres during embryogenesis, but rather function in an epigenetic network that stably inactivates their germline potential.

During this work we also found that LET-418 and CHD-3 are members of two distinct NuRD complexes. Besides LET-418 or CHD-3, these complexes contain LIN-53/RbAp, HDA-1/HDAC and LIN-40/MTA, but not their homologues HDA-2/HDAC, HDA-3/HDAC, EGL-27/MTA nor RBA-1/RbAp. The vertebrate and *Drosophila* NuRD complexes also contain the methyl-CpG-binding protein MBD2/3, which is proposed to mediate an interaction between NuRD and methylated DNA [17]. In *C. elegans*, however, a tagged version of MBD-2 did not interact with the two Mi-2 homologues LET-418 and CHD-3 (results not shown) nor did it co-purify with the LIN-53::TAP containing protein complexes. This suggested that MBD-2 is not a subunit of the NuRD complexes in *C. elegans*. In this context it is interesting to note that no DNA methylation has been found in *C. elegans*, and that MBD-2 was proposed to be part of an ancestral DNA methylation system that was lost in this free living nematode during evolution [40]. Likewise, MBD-2 may have been lost from the *C. elegans* NuRD complexes.

Genes encoding components of the LET-418 containing NuRD complex (*let-418*, *lin-53*, *lin-40*, and *hda-1*) belong to the class B synMuv genes, suggesting that a LET-418 containing NuRD complex is implicated in the synMuv B pathway. Interestingly, *mep-1* is also a synMuv B gene, notwithstanding that MEP-1 is not a stable member of the *C. elegans* NuRD complexes in solution. MEP-1, however, could be member of a protein complex distinct from NuRD and MEC that is involved in vulval development. Alternatively, the MEC complex could also play a role in the synMuv B pathway during vulva formation. A similar situation was found in *Drosophila*, where both, dMec and dNuRD, associate with the promoter of proneural genes of the achaete-scute complex [11].

**Figure 8:**
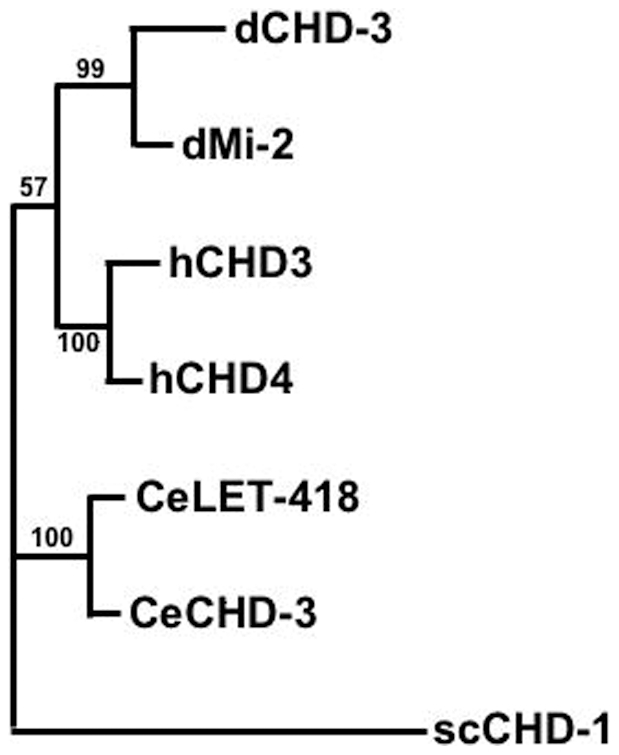
Phylogenetic comparison of the *C. elegans*, *Drosophila* and human Mi-2 orthologues. The regions of the different Mi-2 proteins corresponding to residues 320 to 1076 of the CeLET-418 sequence were used to construct the phylogenetic tree. Numbers refer to bootstrap values supporting particular groupings.

Vertebrates and *Drosophila*, like *C. elegans*, encode two distinct Mi-2 proteins. Our phylogenetic analysis suggests that the gene duplication, giving rise to the second Mi-2 copies must have occurred separately in the three phyla (Figure 8). Gene duplications during evolution often give rise to an essential and a non-essential gene, since the duplicated locus is no longer submitted to selection pressure and therefore is free to change its expression pattern and accumulate otherwise “forbidden” mutations [41], [42]. Changes in the expression pattern and accumulation of new mutations could result in new functions for the duplicated gene copy. In *C. elegans*, *let-418* might have kept its ancient essential functions, whereas *chd-3*, which was not exposed to selection pressure, could have evolved towards new developmental roles. The data presented here suggest that CHD-3 associates with the canonical NuRD components to form a second NuRD complex with new functions.

The two fly Mi-2 orthologues also show some important functional differences. Like LET-418, dMi-2 is an essential protein that is involved in preventing inappropriate expression of developmental transcription programs (reviewed in [43]). This might be achieved by both dMec and dNuRD complexes. The second *Drosophila* Mi-2 orthologue dCHD3 represents a truncated version of dMi-2 that lacks both N-terminal sequences including the first PHD finger and all C-terminal sequences following the ATPase domain. dCHD-3 was shown not to be part of a dNuRD complex and to associate with sites of active transcription [44].

In contrast to the NuRD complexes in *C. elegans* and *Drosophila*, which contain only one orthologue of each protein family, the structure of the vertebrate NuRD complex is more complicated. In addition to either one of the two Mi-2 orthologues Mi-2α or Mi-2β, it contains more than one member of each protein family, i.e. two class I histone deacetylases (HDAC1 and HDAC2), two histone-binding proteins (RbAp46 and RbAp48), one or more metastasis-associated proteins MTA (as well as splice variants of them) and two methyl-binding MBD proteins (reviewed in [5]). The existence of multiple genes encoding similar yet distinct subunits may allow to alter the protein composition of different NuRD complexes, and play an important role in regulating and fine tuning their various cellular functions. Thus, the vertebrate NuRD complex may have gained complexity during evolution not only by duplicating the Mi-2 genes, but also by accumulating several isoforms and variants of the other NuRD components. It will be interesting to see whether different developmental stages or tissues in *C. elegans* use slightly different NuRD and MEC complexes.

## Materials and Methods

### Culture conditions and *C. elegans* strains

All strains were cultured at 20°C, unless otherwise specified, under standard conditions [45]. Wild-type worms were *C. elegans* var. Bristol (N2). Strains used: *chd-3(eh4)X* (FR1156); *lin-53(n833)I lin-15(n767)X* (MT8374); *lin-53(n833)I unc-76(e911)V lin-15(n767)X nls120[gfp::lin-53]* (MT10411); *unc-119(ed3)III Ex[lin-40::gfp*+*unc-119(+)]* (MH1951); *unc-119 (ed3) swIs37[pEXPRlin-53::TAPtag]* (FR932); *dpy-5(e61) lin-53(n833)I swIs37[pEXPRlin-53::TAPtag]* (FR1011); *egl-27(n170)II* (MT170); *unc-29(e1072)I egl-27(n170)II mhEx007[egl-27::gfp]* (KS0024); *lin-15A(n767)X* (MT1806); *lin-15B(n744)X* (MT2495); *lin-35(n745)I* (MT10430); *lin-40(ku285)V* (MH1914); *qIs56 [lag-2::gfp]* (JK2868); *swEx640-1-2[plet-418::VENUS;pchd-3::DsRed2;pRF4]* (FR1111-2-3); *let-418(n3536ts)* (FR843).

### Anti-LET-418 and anti-CHD-3 antibodies

The generation of LET-418 antibodies was described previously [14]. Anti-CHD-3 antibodies were generated by immunizing rabbits with GST-CHD-3 fusion proteins (amino acids 1-267, clone pEJ12). Anti-CHD-3 antiserum was purified by immunodepletion against *chd-3(eh4)* mutant worms. The antibodies recognized a protein of expected size in extracts from wild-type worms, that was absent in extracts from *chd-3(eh4)* animals (see Figure 1B).

### Co-immunoprecipitation

Co-immunoprecipitations were adapted from [14], [46], [47]. For each immunoprecipitation reaction, approximatively 1 mg of protein extract from mixed-stage worms was used, together with 1 µg of antibodies per reaction. Mouse monoclonal Anti-AFP mAb 3E6 (Quantum Biotechnologies) were used to co-precipitate GFP-tagged proteins. To detect GFP-tagged proteins on western blot, monoclonal mouse anti-GFP from (Roche) were used. Rabbit anti-HDA-2 (sc-5551) were from Santa Cruz Biotechnology. Anti-MEP-1, HDA-1 and -3, and LIN-53 antibodies were generous gifts from A. Puoti, Y. Shi and R. Horvitz respectively.

### Construction of LIN-53::TAP-tagged clones

pBS1479 TAP-tag containing vector [48] and pMM016b genomic *unc-119* containing vector [49] were digested with *Hind*III and *Not*I. *unc-119* fragment was ligated into digested pBS1479 to produce pBSunc-119. This vector was digested with *Nco*I, blunted with Klenow enzyme and an RfA cassette of Invitrogen Gateway vector conversion system was ligated. It was subsequently digested with *Hind*III and a PCR amplified *unc-54* 3′UTR with *Hind*III flanking sequences was inserted to produce pDEST-TAPtag. Primers: unc-54 3′UTRl aagcttgtccaattactcttcaacatcc and unc-54 3′UTRr aagcttataaggtattttgtgtgcgg. Promoter and coding sequence of *lin-53* were amplified with Finnzyme Phusion polymerase. Primers: lin-53attB1left ggggacaagtttgtacaaaaaagcaggctgctcaaaatcacgagaatcc and lin-53attB2right ggggaccactttgtacaagaaagctgggtcctgttgtctctctaccacatcg. PCR products were recombined with pDONR201 by Gateway BP recombination to produce pENTR-lin-53. Entry clones were recombined by LR recombination with pDEST-TAPtag to produce pEXPR-LIN-53::TAP.

### Microparticle bombardment

Bombardments were done as previously described [49] except that synchronized worms were grown in liquid culture.

### Protein extraction and tandem affinity purification

About 10 g of worms were grown in liquid culture, washed several times with M9 and incubated for 30 min before two washes with H_2_O. Worms were ground in liquid N_2_ till a thin powder. Same volume of protein extraction buffer (PEB: 50 mM HEPES pH 7.2, 150 mM KCl, 20% Glycerol, 1 mM DTT, 2 mM EDTA, 0.3% NP-40, 1 mM PMSF, Complete Mini EDTA-free (Roche)) was added. The mixture was homogenized at 30000 rpm for 15 sec with a 12 mm Polytron MR 2100 stem and put on ice for 1 min. The procedure was repeated three times. A SLM Aminco 40K French pressure cell was loaded and operated at 10000 psi. The resulting solution was centrifuged for 10 min at 16000 rpm at 4°C in a Type 50Ti rotor. The soluble protein supernatant fraction was kept on ice at 4°C. The pellet was resuspended in a minimal volume of PEB and sonicated on ice with a MSE 150W ultrasonic disintegrator on m power and 2 amp settings for 30 sec and 3 min of pause. The procedure was repeated three times. The suspension was centrifuged for 10 min at 16000 rpm at 4°C in a Type 50Ti rotor. Both fractions were pooled together and centrifuged for 20 min at 38000 rpm at 4°C in a Type 50Ti rotor. TAP was done as described [50], except that NP-40 buffer was replaced by PEB and final eluates were both combined for protein identification by liquid chromatography tandem mass spectrometry (LC/MS/MS). Pellets were either resolved by gel electrophoresis (only one experiment) or directly digested for analysis.

### Enzymatic digestion and mass spectrometry

In-gel digestion was performed according to [51]. Briefly, gel pieces were washed twice with 100 mM NH_4_HCO_3_/50% acetonitrile and once with acetonitrile. Gel pieces were re-hydrated with 15 µl of a trypsin solution (0.1 µg/µl trypsin (trypsin recombinant, Proteomics Grade, Roche) in 25 mM Tris-HCl/2 mM CaCl_2_, pH 8.2) and an additional 15 µl of the same buffer. After incubation overnight at 37°C peptide were extracted twice with 0.1% trifluoroacetic acid/50% acetonitrile, dried, and dissolved in 0.1% formic acid and analyzed by LC/MS/MS on a QTOF Ultima connected on-line to a capillaryHPLC (Waters). Proteins were identified by using ProteinLynx Global Server and Mascot search engines. Precipitated proteins were dissolved in 50 µl trypsin solution (same as above). RapiGest (Waters) was added (5 µl of a 1% solution in water) and digestion was carried out at 37°C for 6 hours. After hydrolysis of RapiGest (according to the RapiGest manual) samples were dried, dissolved in 0.1% formic acid and analyzed by LC/MS/MS on a QTOF Ultima connected on-line to a nanoAcquity UPLC (Waters). Proteins were identified by using ProteinLynx Global Server and Mascot search engines.

### RNAi clones

Constructs used for feeding were from the Ahringer RNAi library. For *hda-3*, a 500 bp fragment of cDNA was amplified by PCR and cloned in the feeding vector pPD129.36 using following primers:

hda3up: 5′-ACGCGTCGACATGAGCCTCCAACACTCGAAATC-3′

hda3down: 5′-CATGCCATGGTGATGCTTCAAAAGCTCCAAAATC-3′

### synMuv scoring

L4 worms were put on RNAi feeding plates at 25°C and their progeny was scored for synMuv phenotype. Animals were scored as syuMuv if one or more ectopic pseudovulvae were observed.

### Immunofluorescence

Synchronized RNAi L1 larvae were fixed according to a modified Finney-Ruvkun fixation protocol [52] and stained with anti-PGL-1 antibodies (generous gift from S. Strome). The presence of ectopic P granule expression was analyzed by microscopy.

### *lag-2::gfp* expression analysis

*lag-2::gfp* embryos were transferred onto *RNAi* plates and incubated at 20°C during about 3 days. Ectopic *lag-2::gfp* expression was analyzed by microscopy.

### Microscopy

Pictures were taken with a Zeiss Axioplan 2 microscope running the AxioVision 4.6 software coupled to a Zeiss AxioCam Color camera.

### Preparation of L1 synchronized population

Synchronized wild-type L4 animals were grown at 25°C on bacteria expressing either *gfp*, *let-418* or *mep-1* dsRNA. Bacteria expressing *gfp* (pPE128.110) were used as reference, since RNAi may induce gene expression changes by itself. Eggs were collected by bleaching gravid adults and allowed to hatch in absence of food at 25°C. Newly hatched L1 larvae were fed on bacteria expressing the different dsRNA for three hours to recover from starvation. Same procedure was used to prepare synchronized wild-type and *chd-3(eh4)* mutant L1 larvae except that worms were grown on NGM plates seeded with OP50 *E. coli* at 25°C.

### RNA isolation

The RNA was isolated from three independent batches of 10 µl of packed worms. To maximize RNA isolation, linear polyacrylamide (GenElute-LPA, Sigma) was used. The aqueous phase was separated with MaXtract High Density 2 ml tubes (Qiagen). The purification of total RNA was performed using RNeasy Mini kit (Qiagen), according to manufacturer protocol, including the on-column DNase digestion to eliminate DNA (Rnase-Free DNase Set, Qiagen).

### Microarray analysis

The labeling and the hybridization were done by Functional Genomics Center Zürich, University/ETH Zürich. The total RNA samples were reverse-transcribed with One-Cycle cDNA Synthesis Kit (Affymetrix Inc, Santa Clara, CA). The cDNA were *in vitro* transcribed in presence of biotinlabeled nucleotides using a IVT Labeling Kit (Affymetrix Inc.). The samples were hybridized to GeneChip® *C. elegans* Genome Arrays containing 22’500 transcripts (Affymetrix) as three biological replicates. The raw data processing was performed using the Affymetrix AGCC software. After hybridization and scanning, the probe cell intensities were calculated and summarized for the respective probe sets by means of the MAS5 algorithm [53]. To compare the expression values of the genes from chip to chip, a global scaling was performed, which resulted in the normalization of the trimmed mean of each chip to target intensity (TGT value). The quality control measures were considered before performing the statistical analysis. Two-group analyses using *T*-test were performed in order to filter out genes with unreliable signal level between replicates (*p*-value >0.01) and save genes with significant signal level between samples (*p*-value <0.01). Gene lists were curated by cross-referencing with WormBase (http://www.wormbase.org, release WS211). Microarrays data is MIAME compliant and the raw data has been submitted to GEO (accession numbers GSE21376, http://www.ncbi.nlm.nih.gov/geo/query/acc.cgi?token=vpidlgqiakwcqbi&acc=GSE21376).

### Functional analysis

Curated gene lists were used as input for FatiGO+ (http://www.babelomics.org) [35]. FatiGO+ version 2.0 was used. FatiGO software reports an unadjusted *P*-value based on a Fischer's exact text and an adjusted *P*-value calculated using the FDR procedure of Benjamini and Hochberg [54].

### quantitative Real-Time PCR (qRT-PCR)

The RNA preparation was performed as described for the microarray protocol, without RNA purification. The cDNAs were synthesized using the QuantiTect Reverse Transcription Kit (QIAGEN) according to the manufacturer protocol. SensiMix *Plus* SYBR (Quantace) were used with Corbett Rotor Gene 6000 machine running the software Rotor gene 6000 v1.7 (Corbett research). All the primers were designed using Primer3 online software (http://www.embnet.sk/cgi-bin/primer3_www.cgi), spanning exon-exon junction; *act-1* and *ama-1* were used as internal control for data normalization. The primers efficiency and specificity were tested by generating a melting curve (70°C and 95°C) and the PCR products were analyzed on agarose gel. For all qRT-PCR analyses, data from triplicate reactions were analyzed using the 2^-ΔΔCt^ method. At least two independent experiments (worm culture and RNA isolation) were used to confirm each gene. Primers list used for qRT-PCR is available in Materials and Methods S1.

### Construction of promoter fusions let-418p::Venus and chd-3p::DsRed2

11 and 9 kb of respectively *let-418* and *chd-3* genomic promoters with about 100 bp of first exon were amplified with Finnzyme Phusion polymerase. Primers: let-418attB1left ggggacaagtttgtacaaaaaagcaggctttccttttgaccttttctgtgac, let-418attB2trcr ggggaccactttgtacaagaaagctgggtcagaagaacgctttcgctcag, chd-3attB1left ggggacaagtttgta caaaaaagcaggctccttacgggcaatcattgag, chd-3attB2trcr ggggaccactttgtacaagaaagctgggtcg ttcttcgccgacatcttcttc. PCR products were recombined with pDONR201 by Invitrogen Gateway BP recombination. pENTR-*let-418p* and pENTR-*chd-3p* were recombined respectively with pDEST-VENUS and pDEST-DsRed2 by LR reaction. 5 ng of pEXPR-*let-418p*::Venus, 5 ng of pEXPR-*chd-3p*::DsRed2, 25 ng of pRF4 and 60 ng of *H. influenzae* sonicated genomic carrier DNA were used to transform N2 worms by microinjection. Three independent stable lines were analyzed by confocal microscopy.

### Confocal microscopy

Images were obtained with a Leica SP5 confocal microscope and analyzed with ImageJ software.

### Phylogenetic tree

The UniProtKB protein sequences Q19815 (LET-418), Q22516 (CHD-3), O97159 (dMi-2), O16102 (dCHD-3), Q12873 (hCHD-3), Q14839 (hCHD-4) AND P32657 (CHD-1 *S.c*.), corresponding to aa positions 320 to 1076 of LET-418 were aligned using the ClustalW algorithm with default parameter values. The phylogenetic tree was constructed using the program protml.exe of the package Phylip v. 3.65 [55], [56]. The PAM model of protein sequence evolution was used with the *S.c.* sequence defined as outgroup; other options were default. The relationship of the yeast sequence to the animal sequences is poorly resolved, but in all analyses two sequences from the same group together irrespective of the phylogenetic method were used (maximum likelihood, parsimony or distance).

## Supporting Information

Materials and Methods S1 Supplementary materials and methods.
(0.05 MB DOC)

Figure S1 Microarray data were validated by qRT-PCR analysis. qRT-PCR analyses were performed on 10 randomly chosen genes found to be deregulated in both *let-418(RNAi)* and *mep-1(RNAi)* larvae. The reactions were done in duplicate using independent batches of cDNA.
(0.30 MB TIF)

Figure S2 qRT-PCR analyses were performed on 6 genes deregulated only in *let-418(RNAi)* (A) and 6 only in *mep-1(RNAi)* (B) larvae. The expression of six of these genes was not affected, suggesting they represent false positive signals on their respective microarrays (*mcm-7*, *Y17G7B.10*, *W07G4.5* and *F09F7.7* for *let-418(RNAi*) microarrays; *ego-1* and *ama-2* for *mep-1(RNAi)* microarrays). The six remaining genes turned out to be deregulated in both, *let-418(RNAi)* and in *mep-1(RNAi)* L1 larvae. They correspond to false negative genes on their respective microarrays and can therefore be added to the pool of genes jointly regulated by LET-418 and MEP-1.
(0.43 MB TIF)

Figure S3 Extracts from *lin-40::gfp* mixed-stage worms were subjected to Superdex 200 gel filtration. Fractions were analyzed by Western blot using specific antibodies as indicated on the left of each panels, molecular weights and fraction numbers are indicated on the top.
(0.47 MB TIF)
