# Supplementary material for: Different Mi-2 Complexes for Various Developmental Functions in Caenorhabditis elegans
Source: PLoS One. 2010 Oct 27;5(10):e13681. doi: 10.1371/journal.pone.0013681 (PMC2965115; doi:10.1371/journal.pone.0013681)
Supplement: Materials and Methods S1 — Supplementary materials and methods. (0.05 MB DOC) [file pone.0013681.s001.doc]

**Supplementary materials and method**

***Gel filtration***

Worms carrying a *lin-40::gfp* transgene were grown at 20°C on NGM plates. Harvested worms were resuspended in lysis buffer [13] and mechanically broken using a combination of polytronic disruption, sonication and micro-pestle homogenization. Cellular debris were pelleted by centrifugation and 1 ml cleared lysate (7 mg of proteins) was applied to a Superdex 200 gel filtration column (10/300 GL, GE Healthcare), and resolved in lysis buffer on an Äkta purifier (GE Healthcare). 250 μl fractions were collected and subjected to SDS/PAGE followed by Western blotting analysis for co-fractionating GFP (Roche), LET-418 and CHD-3 (our antibodies), MEP-1, HDA-1 and LIN-53 proteins (generous gifts from A. Puoti, Y. Shi and R. Horvitz, respectively).

***Primers used for qRT-PCR analysis***

*act-1* left: 5'-GTAAGGATACCTCTCTTGGATTGG-3'

*act-1* right: 5'-ATCCATTGTCGGAAGACCAC-3'

*alh-7* left: 5'-GAGAAGGCCGTCCACTCAG-3'

*alh-7* right: 5'-CTAGCTCGGTTTCACGTTGG-3'

*ama-1* left: 5'-GAAGGTCGCAGGTGGATG-3'

*ama-1* right: 5'-CCATGATTTTTCGCTCCTG-3'

*amt-4* left: 5'-CATCCAGAAATGTCCTTGGAA-3'

*amt-4* right: 5'-TCATTCTGGATTTTCTCCTCTCA-3'

*apc-10* left: 5'-aaaggatgggtgaaaattcc-3'

*apc-10* right: 5'-agcactcgaaaatgacgaac-3'

*bath-36* left: 5'-gatctaccccactcacatgc-3'

*bath-36* right: 5'-ttggtccaaaatcaaaaacg-3'

*B0284.4* left: 5'-GCGCTAATGGAAGCACAGA-3'

*B0284.4* right: 5'- TCGTTTAGCGTCTTGCCTTT-3'

*cyp-13A11* left: 5'-GCTGGGAGCTTTAGCAAATTC-3'

*cyp-13A11* right: 5'-TCATAGTGAAGTGTCCACGTGTC-3'

*deps-1* left: 5'-CCATGCCAGAGACAGAGG-3'

*deps-1* right: 5'-ACGATTCGAATGTTGCAGTC-3'

*egg-3* left: 5'-ggatgctgaattcgatgaag-3'

*egg-3* right: 5'-gacgagcttttctcaaaacg-3'

*ego-1* left: 5'-gcaatccttggaatgtcatc-3'

*ego-1* right: 5'-gctccttcagtcgatttctg-3'

*E01G6.3* left: 5'-TGGATTCAGTAGACATATCGATCC-3'

*E01G6.3* right: 5'-AAAATCGTAACAGTTTTTGATAAGCA-3'

*fbxa-103* left: 5'-tgaagaacgcccttcactac-3'

*fbxa-103* right: 5'-tctcgaaaagctggagacac-3'

*F09F7.7* left: 5'-aattgacgggctcactctc-3'

*F09F7.7* right: 5'-ccgtagtcttgctttcttcg-3'

*F10D7.2* left: 5'-agattctccctttgcctacc-3'

*F10D7.2* right: 5'-tcgagacttttctgctctttg-3'

*him-3* left: 5'-AATAACGGAATCGACACGAA-3'

*him-3* right: 5'-CCGGTGAAGTTTCTTGATTG-3'

*H22K11.2* left: 5'-TGTCAAGAGGAATTCAAGGAAA-3'

*H22K11.2* right: 5'-CAGACTCTCGTAATTTTTCACCA-3'

*mcm-7* left: 5'-gcttcgtctatccacgattg-3'

*mcm-7* right: 5'-tgcgcttctcaattttgttc-3'

*mex-1* left: 5'-tcatcatcatcaacgtcgtc-3'

*mex-1* right: 5'-tgttctctggttttccgaac-3'

*nlp-5* left: 5'-gcatcttttgtggtttcctc-3'

*nlp-5* right: 5'-cctccaagagtgtcaaaacc-3'

*oma-2* left: 5'-ccacttgagactgaggaagc-3'

*oma-2* right: 5'-ctcaaacggactgattggac-3'

*pgl-1* left: 5'-GAAACAGTTGATGTTGGATGG-3'

*pgl-1* right: 5'-CTCGAAAAGTTGAGCGATATG-3'

*puf-3* left: 5'-ttctcgaagtctttgaactcg-3'

*puf-3* right: 5'-ttctcgaagtctttgaactcg-3'

*R09D1.5* left: 5'-TTTGACAAAATCTCCATGCAA-3'

*R09D1.5* right: 5'-CCATATGAATAAAAGCAAAAGACC-3'

*ttr-17* left: 5'-aacaaggaaggagaattcgag-3'

*ttr-17* right: 5'- acatcctggtttggtattgc-3'

*W07G4.5* left: 5'-acttgccgcttgtaatcttg-3'

*W07G4.5* right: 5'-tcgtgcatagctctgatgtc-3'

*Y17G7B.10* left: 5'-tttagcgggaattttgaatg-3'

*Y17G7B.10* right: 5'-ctgttcagttgctgatcgtg-3'

*Y38E10A.14* left: 5'-aacaatcgacagcaagaagc-3'

*Y38E10A.14* right: 5'-gtacttgattgggccagttg-3'

*Y41D4B.16* left: 5'-caaatcggttagggtacagc-3'

*Y41D4B.16* right: 5'-acttgagagcctgatgttgtg-3'
