# Supplementary figures and images for: Different Mi-2 Complexes for Various Developmental Functions in Caenorhabditis elegans
Source: PLoS One. 2010 Oct 27;5(10):e13681. doi: 10.1371/journal.pone.0013681 (PMC2965115; doi:10.1371/journal.pone.0013681)

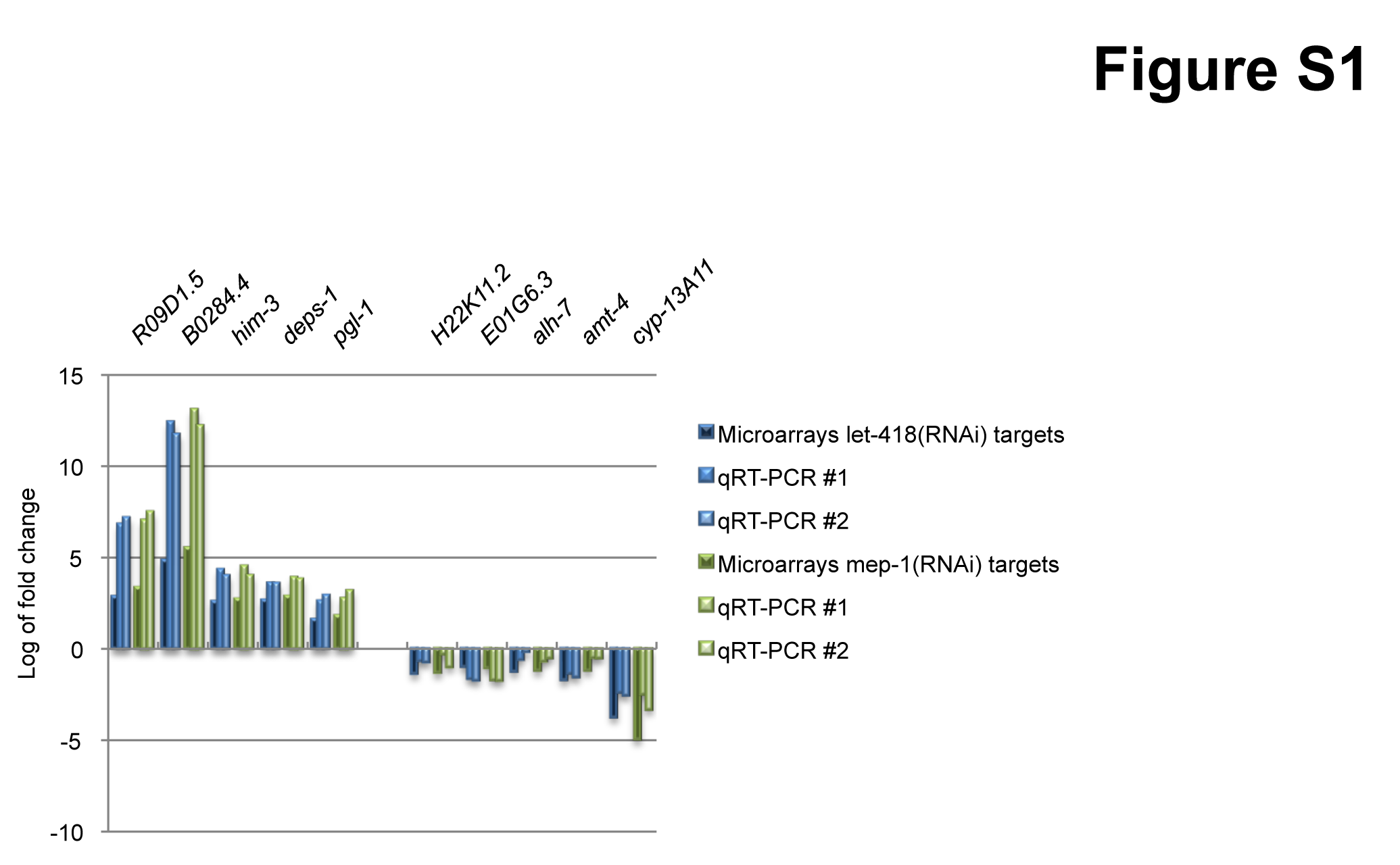

Supplement: Figure S1 — Microarray data were validated by qRT-PCR analysis. qRT-PCR analyses were performed on 10 randomly chosen genes found to be deregulated in both let-418(RNAi) and mep-1(RNAi) larvae. The reactions were done in duplicate using independent batches of cDNA. (0.30 MB TIF) [file pone.0013681.s002.tif]

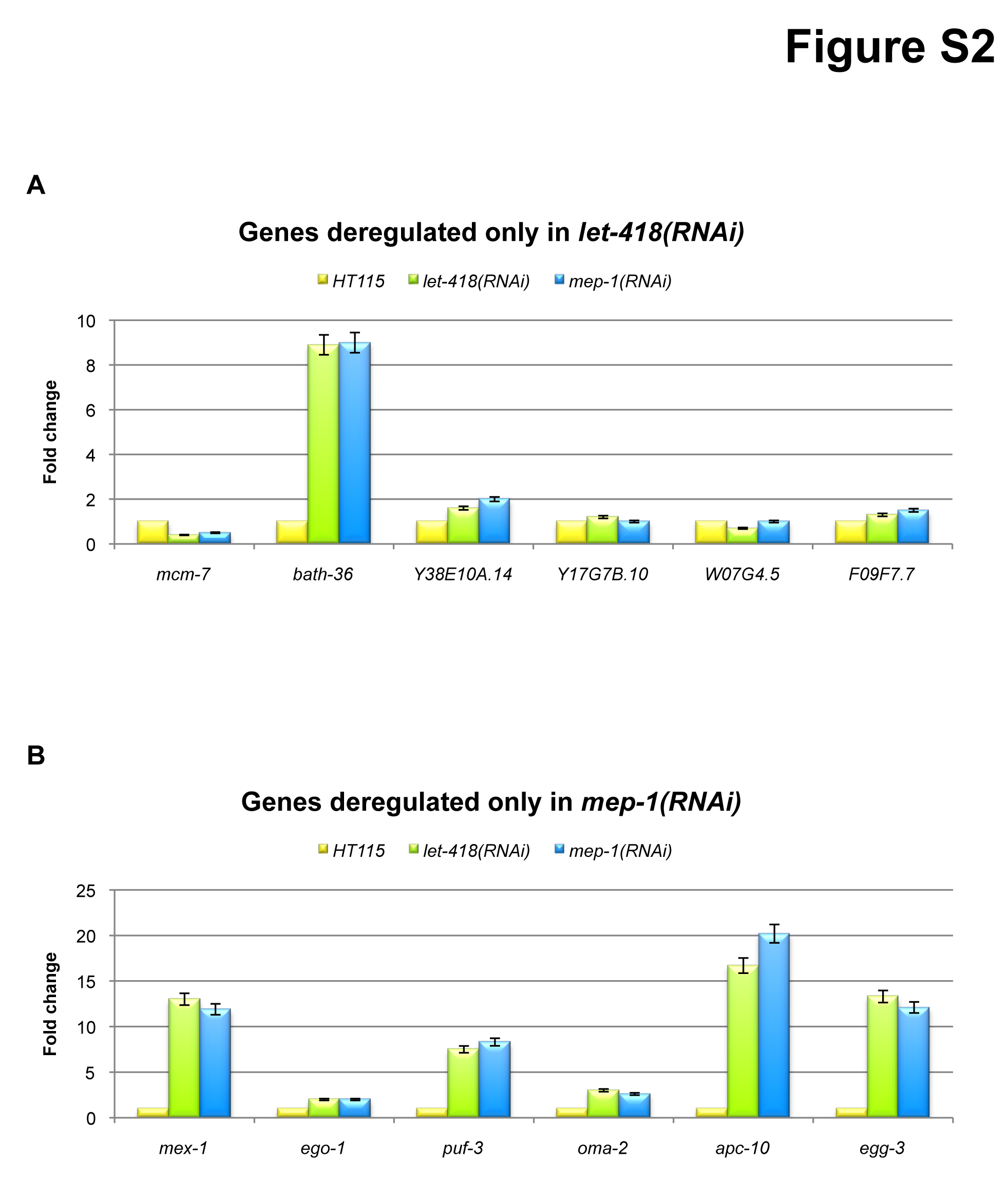

Supplement: Figure S2 — qRT-PCR analyses were performed on 6 genes deregulated only in let-418(RNAi) (A) and 6 only in mep-1(RNAi) (B) larvae. The expression of six of these genes was not affected, suggesting they represent false positive signals on their respective microarrays (mcm-7, Y17G7B.10, W07G4.5 and F09F7.7 for let-418(RNAi) microarrays; ego-1 and ama-2 for mep-1(RNAi) microarrays). The six remaining genes turned out to be deregulated in both, let-418(RNAi) and in mep-1(RNAi) L1 larvae. They correspond to false negative genes on their respective microarrays and can therefore be added to the pool of genes jointly regulated by LET-418 and MEP-1. (0.43 MB TIF) [file pone.0013681.s003.tif]

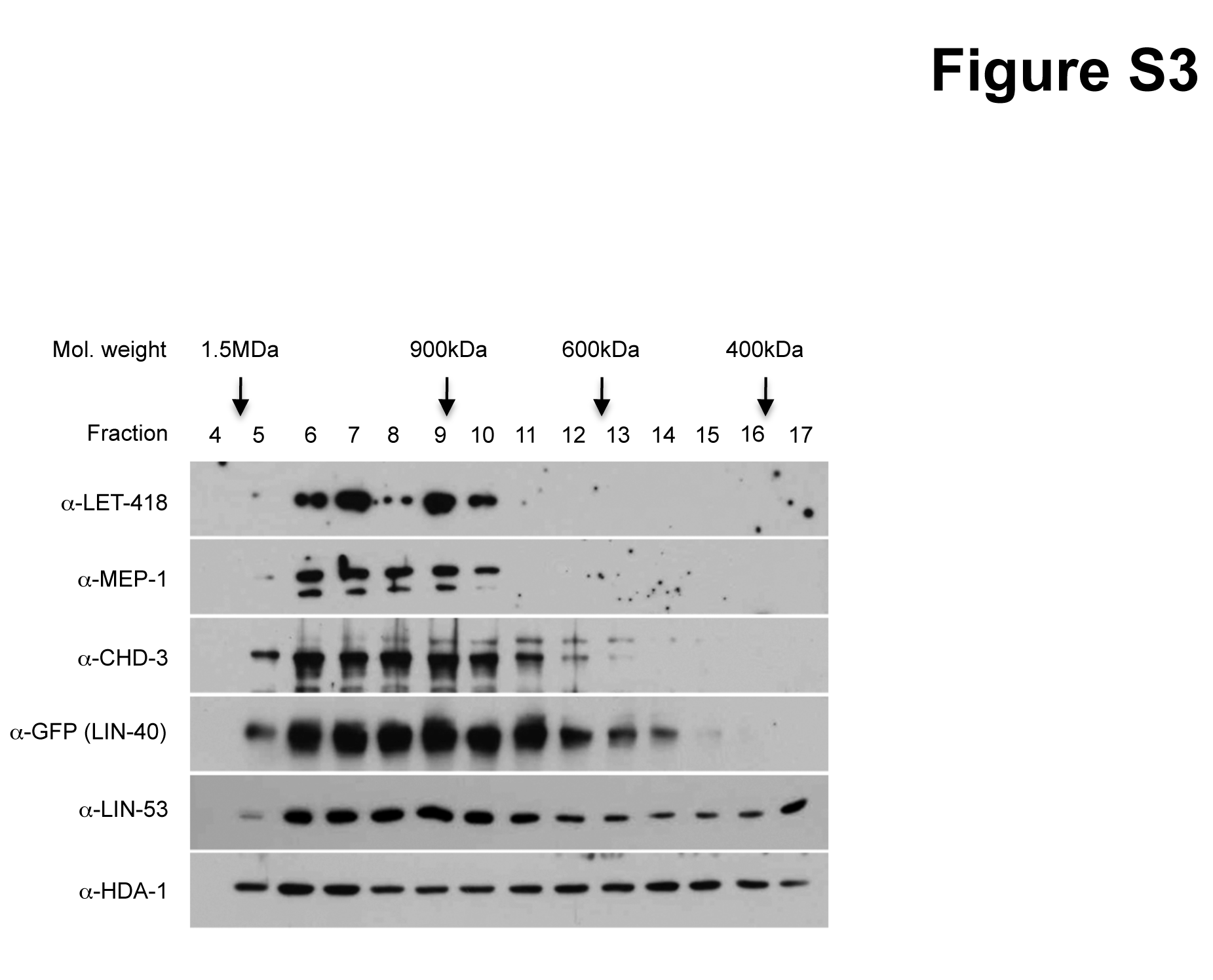

Supplement: Figure S3 — Extracts from lin-40::gfp mixed-stage worms were subjected to Superdex 200 gel filtration. Fractions were analyzed by Western blot using specific antibodies as indicated on the left of each panels, molecular weights and fraction numbers are indicated on the top. (0.47 MB TIF) [file pone.0013681.s004.tif]
